# The Relationship between Physical Health and Psychological Well-Being among Oldest-Old Adults

**DOI:** 10.4061/2011/605041

**Published:** 2011-06-05

**Authors:** Jinmyoung Cho, Peter Martin, Jennifer Margrett, Maurice MacDonald, Leonard W. Poon

**Affiliations:** ^1^Department of Human Development and Family Studies, Iowa State University, Ames, IA 50011, USA; ^2^School of Family Studies and Human Services, Kansas State University, Manhattan, KS 66506, USA; ^3^Institute of Gerontology, University of Georgia, Athens, GA 30602, USA

## Abstract

The purpose of this study was to evaluate the relationship between physical health and psychological well-being among oldest-old adults. Structural equation modeling was performed to examine health influences on psychological well-being among 306 octogenarians and centenarians from the Georgia Centenarian Study. Latent variables were created to reflect subjective health, as measured by self-ratings of health and objective health, as measured by physical health impairment (i.e., health problems, past and present diseases, hospitalization) and biomarkers (i.e., hemoglobin and albumin). Psychological well-being was measured by positive and negative affect. There were significant direct effects of subjective health on affect and significant indirect effects of objective health through subjective health on positive affect and negative affect. Subjective health took the role of a mediator between objective health and psychological well-being. These results highlight the status and perceptions of health as a critical indicator for well-being in extreme old age.

## 1. Introduction

With the unprecedented increase in the number of oldest-old adults, several studies have paid attention to centenarians and their lives exploring factors related to their longevity, such as health, genetic influences, general lifestyle, physical activity, nutrition, and social relationships [1]. Even though many researchers indicate that centenarians have several chronic diseases [2], and health is a significant indicator for psychological well-being among oldest-old adults, only a few studies have focused on health and its impact on psychological well-being in extreme old age. Therefore, there is a need to investigate the association between health and psychological well-being among oldest-old adults.

Usually, *physical health* is the most commonly used index to assess the well-being of individuals. As people grow older, they might perceive that their physical health (e.g., the prevalence rates of chronic conditions) is not as good as it has been in the past. The importance of health among oldest-old adults, especially the prevalence rates of chronic conditions, was shown in a study of Danish centenarians [2]. They found that there were few healthy centenarians and that most Danish centenarians had several common diseases and chronic conditions such as cardiovascular disease (72%), osteoarthritis (54%), hypertension (52%), dementia (51%), and ischemic heart disease (28%). Andersen-Ranberg et al. [2] concluded that it is a challenge to be free from potentially common diseases until the age of 100. This assertion was supported by another centenarian study. After assessing the health history of 424 centenarians, Evert et al. found that even though 19% of centenarians were classified as “escapers” who had reached their 100th birthday without the diagnosis of common age-related diseases, 81% of centenarians were not free from common age-related diseases [3]. Therefore, most oldest-old adults reported chronic health conditions. 

*Subjective health* is “related not only to length of life but also to states of health in the years remaining” [4, p.S315] and serves as one of the most important determinants for psychological well-being in later life. Hoeymans and colleagues noted that subjective health is a valuable and personalized health indicator, specifying one's perception and evaluation of one's own health, based on an interpretation of the objective physical and mental health status, and expectations and comparisons [5–10]. Because individuals' attitudes, motivations, and beliefs influence perceptions of illness and disability, individual differences in subjective health might play an important role for psychological well-being in later life [11]. For instance, as psychological characteristics involve an individual's ability and willingness to adapt to physical change [12], the subjective experience is influenced by various kinds of diseases or illness histories [11, 13].

*Psychological well-being* has been examined as an indicator of successful adaptation during old and very old age [14]. Bradburn considered the subjective assessment of well-being as the balance between positive and negative affect [15]. These two dimensions of well-being may be the origin of psychological well-being [15, 16]. The two types of affect may have different adaptive functions. Negative affect refers to a consequence of maladaptive behavior, whereas positive affect may be considered reinforcement for adaptive or appropriate behavior [15, 16]. Larson summarized previous studies of psychological well-being performed over a 30-year period (1940s–1970s) and noted that the construct is strongly associated with physical health status, functional status, and socio-demographic factors, including occupation, income, educational level, and the degree of social interaction [17, 18]. Hamashima examined previous studies of psychological well-being (specifically, quality of life) in Japan and concluded that it was influenced by physical health and other factors such as age, marital status, occupation, and economic status [18–20].

Based on previous studies, the effect of physical health needs to be considered when accounting for well-being in later life. The importance of physical health for psychological well-being has been reported in a number of studies. Revicki and Mitchell, for example, found that physical health problems were the most important source of life strain among older adults [21]. Physical health can have a major impact on subjective well-being. For instance, Bishop et al. found that poor health was a significant factor associated with lower morale [22]. In addition, there are several studies that have focused on the influence of specific diseases on psychological well-being. For example, positive affect was related to fewer stroke symptoms [23], and low cardiovascular risk was associated not only with better survival but also with better psychological well-being in older adults [24]. These studies all demonstrated that perceived health is associated with objective health [25]. Several studies uncovered the strong relationships between perceived health and long-standing chronic illness, especially among older adults [25–29] and with other health indicators such as number of medications, sick days, or hospitalizations [25, 30–32]. 

As shown in previous studies, there is a close association between objective and subjective assessments of personal health, and this association influences psychological well-being [11]. In other words, individuals' psychological well-being is affected by medical history, current physical symptoms and body sensations, health beliefs and behaviors, and mental and emotional well-being [11, 33]. Additionally, the major factor of subjective health is objective physical health, that is, chronic conditions and disabilities. Many studies have noted the relations between chronic conditions, disabilities, and subjective health [5, 25, 34–38]. Interestingly, Kempen and colleagues observed that health perceptions were most affected by heart conditions, followed by asthma/chronic bronchitis, joint complaints, back problems, and diabetes [35].

Even though a number of studies have suggested a strong association between physical health (objective and subjective) and psychological well-being, many studies only include individuals between the ages of 60 to 80 years, and there is little information about this association for very old age [39]. Therefore, additional research needs to focus on both physical health markers and subjective health predicting psychological well-being in very late life because this time is often characterized by a functional decline or breakdown of the physical and psychological system [39]. The purpose of this study was to assess the association of different aspects (objective and subjective) of physical health and their direct and indirect effect on psychological well-being (i.e., positive and negative affect) in very old adults.

## 2. Method

### 2.1. Participants

The sampling frame of the Georgia Centenarian Study (GCS, Phase III) [40], which provides data for this study, had two components. The first one was to identify the proportion of all residents of skilled nursing facilities (SNFs) and personal care homes (PCHs) in a 44-county area in northern Georgia. Based on census proportions, the project identified residents of SNFs and PCHs. The second recruiting strategy was to use the date-of-birth information in voter registration files to identify community-dwelling residents. Based on these two components and five different characteristics (geographic, age, gender, race, and type of residence) a sample of centenarians and octogenarians was drawn for this study [40].

Obtaining information from oldest-old adults is not always easy or feasible. Especially in old age, individuals' abilities to respond are affected by their physical health, cognitive status, or functional abilities [41]. The different levels of those factors among older adults often lead to the use of proxy ratings of health, functional status, or mental health instead of self-ratings [41–44]. LaRue and colleagues suggested that there was a significant relationship between self and physicians' reports; so self-reports could offer a valid measurement for health assessment in old age [43]. Bassett and colleagues reported that there was a significant correspondence between respondents' and proxy reports on cognitive and mental health [42]. These authors also suggested that self-responses on cognitive and psychological status measures can be substituted with proxy responses when the original informant is unavailable [42]. In addition, several studies also found that proxy information is reliable or less biased when respondents are cognitively impaired or depressed [44–46]. Rodgers and Herzog, for example, indicated that there has been a general consensus among researchers that proxy respondents should be used in research focusing on oldest-old adults to avoid biasing the data compared to healthy elderly [47]. Therefore, based on these arguments, using proxies data might be helpful not only to substitute for insufficient information of self-reports but also to have different viewpoints of psychological well-being among oldest-old adults. Therefore, the information in this paper is based on proxy information.

Proxy informants were selected in the following fashion: first close family such as spouses or children was considered as proxies. If more than one child was alive, the oldest-old adults nominated a proxy, or in the case of cognitive impairment, a contacted child made the decision about who could provide the most accurate information. Other relatives served as proxies if no children were alive or available or if so nominated by the participant. If no other relatives were alive or available, friends, neighbors, nurses, clergy, or other knowledgeable person also served as proxies. Most of the proxy informants (59.4%) were adult children. Additional proxies included nieces and nephews (10.0%), granddaughters (7.7%), and miscellaneous informants, such as spouses, siblings, or friends (22.9%). 

This study included 306 community-dwelling and institutionalized oldest-old adults aged from 80 to 100 (mean age was 96.55 years). In this study, 79.4% of the participants were women and 75% of participants rated their health as good or excellent. A summary of demographic characteristics is presented in Table 1.

### 2.2. Measures

#### 2.2.1. Physical Health Impairments

Physical health impairments were measured using items with several indicators: past and current diseases, health conditions, and hospitalization. Past and current diseases were assessed with a comprehensive list of diseases such as congestive heart failure, myocardial infarction, and high blood pressure. Health conditions were assessed with a variety of health problems such as chest discomfort, numbness, arthritis, and dizziness. Lastly, hospitalization was accessed with any recent or lifetime hospitalization. Higher scores reflect more health problems, more diseases, and more hospitalizations.

#### 2.2.2. Biomarkers

Biomarkers included hemoglobin and albumin, which were assessed with a blood draw. Higher scores indicate higher levels of hemoglobin and albumin.

#### 2.2.3. Subjectively Perceived Health

The subjective perception of health was comprised of two questions [48] with an original internal consistency coefficient *α* = 0.74. Proxies were asked: “How would you rate his/her overall health at the present time—excellent, good, fair, or poor?” and was scaled so that 0 = poor to 3 = excellent. The other question was “How much do his/her health troubles stand in the way of his/her doing the things he/she wants to do?” and was scaled so that 0 = a great deal to 2 = not at all. Internal consistency for the proxy ratings of our participants was *α* = 0.56. Physical health was scored so that higher scores indicated higher levels of physical health.

#### 2.2.4. Psychological Well-Being

Psychological well-being was assessed with the Bradburn Affect Balance Scale [15]. The scale consists of two dimensions: positive affect and negative affect. Five positive affect items (*α* = 0.80) and five negative affect items (*α* = 0.80) from proxy reports were used in this study. Proxies were asked to rate centenarians with the following statements for positive affect. During the past two weeks, (1) Did he/she ever feel pleased about having accomplished something? (2) Did he/she ever feel proud because someone complimented him/her on something he/she had done? (3) Did he/she ever feel particularly excited or interested in something? (4) Did he/she ever feel that things were going his/her way? (5) Did he/she ever feel on top of the world? For negative affect, the following statements were asked. (1) Did he/she ever feel depressed and very unhappy? (2) Did he/she ever feel vaguely uneasy? (3) Did he/she ever feel bored? (4) Did he/she ever feel so restless that he/she could not sit long in a chair? (5) Did he/she ever feel very lonely or remote from other people? Ratings were used with a four-point Likert scale: 1 = not at all, 2 = once, 3 = several times, and 4 = often. Higher scores for positive affect indicated better well-being, while higher scores for negative affect indicated lower well-being.

### 2.3. Plan of Analysis

#### 2.3.1. Confirmatory Factor Analysis (CFA)

Confirmatory factor analyses using LISREL 8.71 [49] established the fit of subjective health, objective health, and psychological well-being to the corresponding constructs in this study. Maximum-likelihood estimation was used. The results are summarized in Table 2. In terms of the psychological well-being measure, positive affect and negative affect were initially tested in relation to a model composed of five indicators for each construct. However, the lowest loadings of each construct were dropped after conducting an item analysis, and the model specified three indicators for each affect construct. All the loadings of each factor were significant (Table 2).

#### 2.3.2. Structural Equation Modeling (SEM)

Structural equation modeling was used to test the relationship between subjective health, objective health, and psychological well-being with LISREL 8.71.

## 3. Results

Three different models were tested to examine the relationship between physical health and psychological well-being (Table 3). Model 1 is the measurement model for objective health, subjective health, and psychological well-being and no relationship among physical health impairments, biomarkers, subjective health, positive affect, and negative affect was hypothesized, *χ* ^2^  (df = 78) = 111.19, *P* < .05, CFI = 0.94, TLI (NNFI) = 0.93, and RMSEA = 0.06. Model 2 investigated the relationship between objective health and psychological well-being through subjective health. Model 2 yielded a better fit in comparison to Model 1, *χ* ^2^  (df = 72) = 55.48, *P* = .93, *χ* ^2^ diff (6) = 55.71, *P* < .001, CFI = 1.00, TLI (NNFI) = 1.03, and RMSEA = 0.00 (Table 3). Model 3 tested the full model of direct effects of objective health (physical health impairments, biomarkers) and subjective health on psychological well-being. All possible relationships between health and psychological well-being were hypothesized to be correlated. In comparison to Model 1, Model 3 yielded a better model fit, *χ* ^2^  (df = 68) = 53.75, *P* = .90, *χ* ^2^ diff (10) = 57.74, *P* < .001, CFI = 1.00, TLI (NNFI) = 1.03, and RMSEA = 0.00 (Table 3). However, Model 3 was not significantly better in comparison to Model 2, *χ* ^2^ diff (4) = 1.73, and *P* = .79. Therefore, Model 2, the more parsimonious model, was selected as the best fitting model (Table 3).

**Figure 1:**
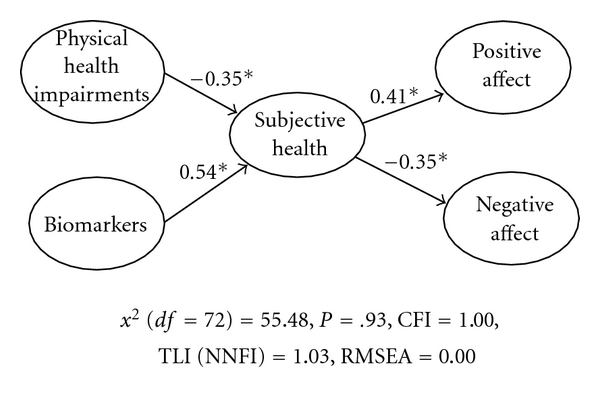
The latent variable relationship between physical health impairment and psychological well-being. *Note.* Path coefficients are standardized parameter estimates and direct loadings are displayed in solid lines. *P < .05.

Based on Model 2, the latent variable relationships between health and psychological well-being were inspected (Figure 1). This model examined the mediating effect of subjective health between objective health (i.e., physical health impairments and biomarkers) and psychological well-being (i.e., positive affect and negative affect). Paths between physical health impairments, biomarkers, subjective health, positive affect, and negative affect were investigated (Figure 1).

There were several significant direct effects in Model 2. In terms of physical health, physical health impairments were found to have a significant negative direct effect on subjective health (*β* = −0.35, *P* < .05). Biomarkers had a significant positive effect on subjective health (*β* = 0.54, *P* < .05). Second, subjective health had significant direct effects on positive and negative affect. In other words, subjective health was found to be significantly associated with positive affect, *β* = 0.41, *P* < .05, and to have a significant negative association with negative affect, *β* = −0.35, *P* < .05. Third, there was an indirect effect of objective health on psychological well-being. Specifically, physical health impairments had significant indirect effects on positive affect, *β* = −0.14, *P* < .05, and negative affect, *β* = 0.12, *P* < .05. Biomarkers also had significant indirect effects on positive, *β* = 0.22, *P* < .05, and negative affect, *β* = −0.19, *P* < .05. In other words, there was a mediating effect of subjective health between physical health and positive affect and negative affect.

## 4. Discussion

The purpose of this study was to highlight the way in which physical health influences psychological well-being among oldest-old adults. Two potentially important findings emerged through structural equation modeling. First, the analysis suggests that subjective health was strongly associated with psychological well-being (e.g., affect) among oldest-old adults. Second, the results further revealed that physical health impairments and biomarkers had independent direct effects on subjective health and they had an indirect association with psychological well-being among oldest-old adults. 

There are a couple of reasons why these results emerging from this study are noteworthy. To begin with, the conclusions are based on data that were gathered from an oldest-old population. In general, physical health is recognized as one of the most important indicators of quality of life in later life. Even though the importance of studying very old populations has been noted repeatedly, few studies (e.g., [49]) have explored the relationship of health with psychological well-being for very old persons. In addition, specification of physical health by different assessments such as physical health impairment, biomarkers, and subjective health helps underscore the importance of including different aspects (objective and subjective) of physical health and the different role they play for psychological well-being in very late life. Finally, the findings from this study were based on proxy information. This is important because many researchers indicate that it is less reliable to use proxy information due to proxy bias. However, consistent with earlier studies [44–47], the results of this study contribute to the argument that information from proxies could provide sufficient information and unique perspectives of psychological well-being among oldest-old adults. 

The findings of this study were supported by previous studies. First, objective aspects of physical health (e.g., physical impairment, biomarkers) had an independent direct effect on subjective health. Earlier studies showed that chronic diseases were significantly associated with subjective health or perceived health status [44–47]. For example, Jylhä and colleagues found that different factors were associated with self-rated health for different age groups [27]. The results showed that the number of chronic diseases such as high blood pressure was the strongest predictor of self-rated health among older adults aged 70 to 79 [27]. This underlines the importance of physical health impairment for perception of health among old and oldest-old population. Furthermore, the results of this study are consistent with the findings of recent studies that biomarker assessments are used in combination with behavioral and social aspects related to individuals' health and well-being [50–52]. Jylhä et al. showed a significant association between biomarkers and self-rated health. Interestingly, lower levels of hemoglobin were significantly associated with fair or poor self-rated health [53]. Another result of this study confirmed previous findings that reported a significant association between psychological well-being and subjective health among oldest-old adults [6–11]. Perhaps the most noteworthy finding of this study was the significant indirect effect of physical health on the psychological well-being among oldest-old adults. This is consistent with other studies reporting that those higher in positive affect reported fewer severe disease symptoms, and those higher in negative affect reported more severe ones (e.g., [54]). This finding is supported by the work of Temane and Wissing [55] who showed that the subjective perception of health mediated the relationship between individual context such as physical health and psychological well-being [55]. Therefore, the results of our study lead us to conclude that perceived health takes the role of an important mediator between physical health and psychological well-being.

Even though this study made significant contributions to the literature by linking two perspectives of health and psychological well-being, there are also several limitations of the present study. The sample of this study was from only one geographic area of the United States. Other oldest-old adults in different regions might present different patterns in the relationship between physical health and psychological well-being. Second, although physical health was assessed with the number of present and past diseases, those indicators were examined with a cross-sectional design. Therefore, causal inferences on the relationship between health and well-being cannot be made. Finally, even though most of the indicators were examined by proxy ratings and quite a few papers have demonstrated that proxy informants are reliable and substitutable for self-rated reports to use, we need to consider that disagreement on psychological aspects might result in differences of proxy—and centenarians' self-ratings. 

In spite of these limitations, the results of this study support the notion that health, subjective and objective, is an essential factor for psychological well-being in later life. Fewer problems with physical health (i.e., number of diseases, health problems, and hospitalization) and more favorable readings of hemoglobin and albumin influence perceptions of health, and this has a positive effect on positive affect and a negative effect on negative affect among very old persons. Even though physical health problems are common among octogenarians and centenarians, the results confirm that both physical and psychological well-beings are critical factors at the very end of the human life span.

## Figures and Tables

**Table 1:** Summary of demographic characteristics.

Demographic characteristics	*n*	%
Age		
Octogenarian (*M* = 84.58)	72	23.5
Centenarian (*M* = 100.23)	234	76.5
Gender		
Female	243	79.4
Male	63	20.6
Type of residence		
Private home/apartment	165	54.1
Personal care	48	15.7
Nursing home	92	30.2
Ethnicity		
White/Caucasian	240	78.4
Black/African American	66	21.6
Education		
Less than high school complete	99	34.1
High school diploma	61	21.0
GED/some college	67	23.1
College/graduate degree	63	21.7
Subjective health		
Poor	10	3.3
Fair	69	22.7
Good	148	48.7
Excellent	77	25.3

**Table 2:** Factor loadings in confirmatory factor of health and psychological well-being.

	Physical health impairments	Biomarkers	Subjective health	Positive affect	Negative affect
Past disease	0.89	—	—	—	—
Current disease	0.55				
Health problem	0.39				
Hospitalization	0.35				
Hemoglobin	—	0.78	—	—	—
Albumin		0.43			
Self-rated overall health	—	—	0.72	—	—
Self-rated health problem			0.63		
Pleased	—	—	—	0.73	—
Proud				0.70	
Excited/interested				0.76	
Depressed	—	—	—	—	0.77
Vaguely uneasy					0.76
Bored					0.70

*Note.* All factor loadings are standardized parameter estimates.

**Table 3:** Fit indices for nested sequence of cross-sectional models.

Model	*χ* ^2^	df	*χ* ^ 2^ diff	CFI	TLI	RMSEA
(1) Measurement model	111.19	78		0.94	0.93	0.06
(2) Health and psychological well-being relation model	55.48			1.00	1.03	0.00
Difference between model 2 and model 1		72	55.71***			
(3) Fully recursive model	53.75	68		1.00	1.03	0.00
Difference between model 3 and model 2			1.73			
(4) Null model	562.57					

***P < .001.
